# Nutritional Supplementation Combined with Exercise for Musculoskeletal Health in Women: A Systematic Review and Meta-Analysis Evaluating Proteins, Amino Acids, and Creatine across Reproductive Stages

**DOI:** 10.7150/ijms.130435

**Published:** 2026-04-16

**Authors:** Kee-Hsin Chen, Tzu-Pei Yeh, Shu-Cheng Lin, Pi-Ju Liu, Dorothy Bai, I-Hui Chen

**Affiliations:** 1 Post-Baccalaureate Program in Nursing, College of Nursing, Taipei Medical University, Taipei, Taiwan.; 2 Cochrane Taiwan, Taipei Medical University, Taipei, Taiwan.; 3 Department of Nursing, Wan Fang Hospital, Taipei Medical University, Taipei, Taiwan.; 4 Evidence-based Knowledge Translation Center, Wan Fang Hospital, Taipei Medical University, Taipei, Taiwan.; 5 School of Nursing, China Medical University, 100 Jingmao Rd., Sec. 1, Beitun Dist., Taichung 406040, Taiwan.; 6 Department of Nursing, China Medical University Hospital, 2 Yude Rd., North Dist., Taichung 404327, Taiwan.; 7 Department of Physical Education, Health, and Recreation, Teachers College, National Chiayi University, 85, Wen Long Vil., Minxiong Township, Chiayi County 621302, Taiwan.; 8 School of Nursing, Center on Aging and the Life Course, Purdue University, 502 North University Street, West Lafayette, IN 47907, USA.; 9 School of Gerontology and Long-Term Care, College of Nursing, Taipei Medical University, 250 Wuxing St, Xinyi District, Taipei 11031, Taiwan.; 10 School of Nursing, College of Nursing, Taipei Medical University, 250 Wuxing St, Xinyi District, Taipei 11031, Taiwan.

**Keywords:** exercise, menopause, muscle strength, osteosarcopenia, nutritional supplementation, sarcopenia

## Abstract

**Background:**

Women experience progressive musculoskeletal deterioration across reproductive stages, with accelerated changes during the menopausal transition. Exercise provides established benefits, but the additive effects of nutritional supplementation strategies, including proteins, amino acids, and related compounds such as creatine, remain uncertain. Previous systematic reviews focused on older adults aged 65 years and above, leaving a knowledge gap regarding women facing unique metabolic challenges related to reproductive hormones. Therefore, this systematic review and meta-analysis was conducted to evaluate whether nutritional supplementation combined with exercise enhances muscle mass, strength, and bone health outcomes in women across reproductive stages, with particular attention to the menopausal transition.

**Methods:**

Eight databases were searched from inception to July 2025: CINAHL, ClinicalTrials.gov, Cochrane Central Register of Controlled Trials, Embase, PsycINFO, MEDLINE/PubMed, Scopus, and Web of Science. We included randomized controlled trials examining nutritional supplementation combined with exercise in women across reproductive stages. Interventions comprised whole proteins, amino acids, amino acid derivatives, or protein-derived peptides administered with structured exercise programs. Comparators included exercise alone or a placebo plus exercise. Primary outcomes were muscle mass measures. Secondary outcomes included muscle strength, bone health parameters, body composition, and adverse events. Random-effects meta-analyses calculated Hedges' g with 95% confidence intervals (CIs).

**Results:**

Fourteen trials including 763 women across reproductive stages met the inclusion criteria, which evaluated whole-protein supplements (five studies), amino acids and derivatives (six studies), and creatine monohydrate (three studies). The combined intervention showed no significant effects on muscle mass measures: skeletal muscle mass (g=0.065, 95% CI: -0.353 to 0.482, *p*=0.762), appendicular lean mass (g=0.197, 95% CI: -0.177 to 0.571, *p*=0.302), or fat-free mass (g=0.069, 95% CI: -0.110 to 0.249, *p*=0.447). Significant improvements occurred in bench press (g=0.279, 95% CI: 0.008 to 0.550, *p*=0.043) and handgrip strength (g=0.412, 95% CI: 0.039 to 0.786, *p*=0.031). No significant effects emerged for bone mineral content (g=0.195, 95% CI: -0.281 to 0.671, *p*=0.421) or bone mineral density (g=0.087, 95% CI: -0.129 to 0.303, *p*=0.430). No increase in adverse events was observed.

**Conclusions:**

Current evidence does not support robust additive effects of nutritional supplementation on muscle mass or bone health when combined with exercise in women across reproductive stages. Selective upper-body strength improvements were observed, particularly in studies using creatine supplementation. Exercise alone provides reliable musculoskeletal health benefits. Given the heterogeneity of supplement types examined, future research should employ longer intervention durations (≥12 months) with site-specific bone measurements and conduct head-to-head comparisons of specific supplement types.

## Introduction

Musculoskeletal health represents a critical concern across women's reproductive lifespan, with the menopausal transition marking a period of accelerated deterioration. While premenopausal women demonstrate relatively stable muscle mass and bone density, the perimenopausal period initiates progressive changes that accelerate during the first postmenopausal decade 1,2. Women across the menopausal transition experience muscle mass declines of 0.5%-1.0% annually, substantially exceeding the 0.3%-0.8% observed in age-matched men 3,4. Concurrently, bone loss accelerates to 1%-3% per year during the first 5 postmenopausal years, compared to 0.5%-1% annually in premenopausal women 5-8. However, musculoskeletal health optimization begins before menopause, as preventive interventions implemented during the premenopausal years may prove more effective than interventions after significant deterioration occurs 9. With over 1.2 billion women expected to be menopausal or postmenopausal by 2030 globally, developing effective interventions that span reproductive stages has become a public health priority 10.

This dual deterioration translates into meaningful functional declines including decreased walking speed, impaired balance, reduced capacity for activities of daily living, and increased fall risks 11. The consequences extend beyond physical function, with over 25% of women reporting substantial limitations during the menopausal transition that affect independence and quality of life 12,13.

The relationship between muscle and bone health is particularly relevant for women during the menopausal transition. Sarcopenia increases fall risks through compromised balance and slower protective reflexes, while also reducing the mechanical loading (muscle forces applied to the bone during contraction) necessary for maintaining bone health. Both muscle strength--particularly lower-extremity strength for mobility and balance, and upper-extremity strength for daily activities--and muscle mass contribute to this mechanical loading. Emerging evidence has demonstrated that sarcopenia and osteoporosis frequently co-occur as osteosarcopenia, with shared pathophysiological mechanisms including an estrogen deficiency, increased inflammatory markers, and altered calcium-protein metabolism 14. This interconnected deterioration suggests that interventions targeting multiple aspects of musculoskeletal health--muscle mass, strength, and bone density--may provide benefits for fracture prevention in women across the menopausal transition 15.

Exercise, particularly progressive resistance training, has demonstrated clear benefits for muscle mass, strength, and overall musculoskeletal health in women during the menopausal transition 16,17. For bone health, high-intensity progressive resistance training combined with impact exercises has shown efficacy in maintaining or modestly improving bone mineral density (BMD) at clinically relevant sites in postmenopausal women with low bone mass 18,19. These exercise-induced benefits occur through multiple mechanisms including enhanced muscle protein synthesis, improved neuromuscular coordination, increased growth factor production, and mechanical loading that stimulates bone remodeling 20,21.

Despite the established benefits of exercise alone, there is a compelling mechanistic rationale for adding nutritional supplementation. An estrogen deficiency impairs muscle protein synthesis through reduced mechanistic target of rapamycin complex 1 signaling and increased myostatin expression, while simultaneously increasing bone resorption through enhanced osteoclast activity and reduced osteoblast function 22,23. Concurrently, aging-related anabolic resistance reduces muscle responsiveness to dietary protein, a phenomenon well-documented in older adults that may be exacerbated by hormonal changes 24. Strategic protein supplementation, particularly when timed appropriately around exercise, can theoretically enhance post-exercise muscle protein synthesis rates and support recovery processes essential for both muscle adaptation and bone formation 25-27.

The potential additive effects of protein supplementation with exercise were demonstrated in older adult populations (≥65 years), with recent systematic reviews, including by Yoshimura et al. 28 who reported benefits for muscle mass and strength when protein supplementation was combined with resistance training. However, it remains unclear whether these benefits translate to women during the menopausal transition, who face unique metabolic challenges from an estrogen deficiency that differ from age-related changes alone.

To our knowledge, no systematic reviews have comprehensively evaluated nutritional supplementation strategies, including whole proteins, amino acids, and related compounds such as creatine, combined with exercise across women's reproductive stages. Previous systematic reviews predominantly focused on older adults aged 65 years and above 28-31, examining narrowly defined whole-protein supplements. This represents a significant knowledge gap for several reasons. First, women in their 40s and 50s experiencing the menopausal transition have different metabolic profiles, activity levels, and recovery capacities compared to adults over 65 years 32, yet musculoskeletal health concerns are not confined to the postmenopausal period. Second, the hormonal environment during perimenopause and early menopause creates unique challenges for protein metabolism that may require different supplementation strategies than those effective in older adults 22,24. Third, diverse supplement types including creatine monohydrate—an amino acid derivative endogenously synthesized from glycine, arginine, and methionine 33,34—and individual amino acids are frequently investigated for enhancing exercise adaptations 35-39, yet their comparative effectiveness in women remains unclear. Although creatine primarily functions through phosphocreatine-mediated energy metabolism rather than serving as a direct protein building block, we classify it as an amino acid derivative based on its biosynthetic origin and its frequent co-investigation alongside protein and amino acid supplements in exercise nutrition research 25,33-38,40. We acknowledge that these compounds differ mechanistically—while whole proteins provide amino acid substrates for muscle protein synthesis, creatine primarily enhances phosphocreatine stores and ATP regeneration 33-36. However, from a pragmatic clinical perspective, these supplements are frequently considered as complementary strategies within comprehensive nutritional approaches to support exercise adaptations 38. Our inclusive approach aimed to capture the breadth of evidence regarding nutritional interventions studied in combination with exercise, recognizing that this heterogeneity limits mechanistic interpretability but enhances real-world applicability. Finally, understanding whether interventions should differ across reproductive stages versus employing consistent strategies throughout the lifespan has important implications for clinical practice and public health policies 41.

To address these knowledge gaps, we conducted this systematic review and meta-analysis examining nutritional supplementation combined with exercise for musculoskeletal health in women across reproductive stages, with particular attention to the menopausal transition as a critical period of accelerated change. Our primary objectives were to quantify treatment effect magnitudes on muscle mass measures, as these directly relate to sarcopenia prevention. Secondary objectives included evaluating effects on muscle strength parameters relevant to functional independence, bone health outcomes essential for fracture prevention, and body composition measures important for metabolic health. By employing an inclusive approach to both participant selection and supplement type, we aimed to capture the breadth of evidence regarding nutritional strategies to enhance exercise adaptations in women, thereby informing clinical practice and identifying priorities for future research. We recognize that pooling mechanistically distinct interventions limits causal inferences about specific supplement mechanisms, but provides valuable evidence regarding the overall state of research in this population.

## Methods

### Protocol and registration

This systematic review and meta-analysis were conducted in accordance with the Preferred Reporting Items for Systematic Reviews and Meta-Analyses guidelines 42. The protocol was registered in PROSPERO (registration no.: CRD420251110761). Two modifications were made to the original registered protocol during the conduct of this review. First, secondary outcomes including muscle strength, bone health parameters, body composition measures, and adverse events were added because the majority of included trials reported these outcomes alongside the primary muscle mass measures; their inclusion provides a more comprehensive and clinically relevant evaluation of musculoskeletal health. Second, certainty of the evidence was assessed using the GRADE approach, which was not prespecified but was incorporated to enhance the interpretability and clinical utility of our findings, consistent with current recommendations for systematic reviews 43. These protocol deviations were transparently reported in accordance with PRISMA 2020 guidelines 42.

### Eligibility criteria

Studies were included based on predefined PICOS (population, intervention, comparison, outcomes, and study) criteria. We included randomized controlled trials (RCTs) examining the effects of nutritional supplementation combined with exercise programs in women across reproductive stages. Eligible interventions comprised whole-protein supplements of any type or dose, individual amino acids including branched-chain amino acids and leucine, amino acid derivatives such as creatine and L-citrulline, or protein-derived peptides administered concurrently with structured exercise programs including resistance training, aerobic exercise, or combined modalities. This inclusive approach was adopted to comprehensively capture nutritional strategies investigated to enhance exercise adaptations in women. We acknowledge that these compounds are mechanistically distinct: whole proteins provide complete amino acid profiles for muscle protein synthesis, isolated amino acids (e.g., leucine) serve as specific anabolic signals, and creatine—although synthesized from amino acids (glycine, arginine, and methionine)—primarily functions through phosphocreatine-mediated energy metabolism rather than as a protein building block 33-36. Despite these mechanistic differences, we included all of these interventions because: (1) they are frequently studied together in exercise nutrition research as complementary strategies 25,33,35-38,40; (2) from a clinical practice perspective, women and healthcare providers often consider these as part of comprehensive nutritional approaches to musculoskeletal health 38; and (3) excluding mechanistically distinct supplements would fragment the evidence base and obscure the overall state of research in this population. We addressed the implications of this heterogeneity in our limitations and interpreted pooled estimates with appropriate caution. This inclusive approach to supplementation use was adopted because these compounds share common metabolic pathways related to muscle protein synthesis and energy metabolism 40, and are frequently investigated as strategies to enhance exercise adaptations 25,33,35-38.

Study populations included women classified as premenopausal, perimenopausal, menopausal, or postmenopausal according to original study definitions. This broad inclusion criterion was chosen because musculoskeletal health optimization is relevant across all reproductive stages 44, with the menopausal transition representing a period of accelerated but not exclusive change 1,2,5-8. Comparators included exercise alone or placebo plus exercise. Primary outcomes were quantitative measures of muscle mass including lean body mass, appendicular lean mass, skeletal muscle mass, and fat-free mass measured at the baseline and post-intervention. Secondary outcomes included muscle strength parameters, bone health measures including bone mineral density (BMD) and bone mineral content at any skeletal site, body composition indicators, and adverse events. Studies were excluded if they involved participants with hereditary muscle disorders or conditions associated with muscle wasting (cancer, HIV/AIDS, or chronic kidney disease), or had a non-randomized study design. Only English-language publications were considered for inclusion.

### Search strategy

We conducted a comprehensive literature search across eight electronic databases from inception through July 2025: CINAHL, ClinicalTrials.gov, Cochrane Central Register of Controlled Trials, Embase, PsycINFO, MEDLINE/PubMed, Scopus, and Web of Science. The comprehensive search strategy combined controlled vocabulary (e.g., MeSH terms, Emtree terms, CINAHL headings) and free-text keywords using Boolean operators, tailored to each database's syntax. The complete search strategies for all eight databases are provided in Supplementary Table S1. An example search for MEDLINE included the following: (menopause OR postmenopaus OR perimenopaus^*^) AND (protein^*^ OR amino acid^*^ OR supplement^*^) AND (exercise OR resistance training OR physical activity) AND (muscle mass OR lean mass OR sarcopenia OR bone density). Reference lists of included studies and relevant reviews were manually searched to identify additional eligible studies.

### Study selection and data extraction

Following duplicate removal, two independent reviewers (KHC and IHC) screened titles and abstracts against eligibility criteria. Full-text articles were retrieved for potentially eligible studies and independently assessed for final inclusion. Disagreements were resolved through discussion. Data were independently extracted by two reviewers (KHC and IHC) using a standardized, pilot-tested form. Extracted variables included study characteristics (author, year, and country); participant characteristics (sample size, age, menopausal status, and health condition); intervention parameters (protein type, dose, timing, exercise modality, duration, and adherence); comparison group details (type of control intervention, dose, exercise modality, duration, and adherence); outcome measures (muscle mass assessment method, and specific measures and units); and results (pre-intervention and post-intervention values, within-group changes, between-group mean differences, and statistical significance). When reported data were incomplete or required clarification, study authors were contacted via email with a maximum of two contact attempts over a 4-week period.

### Risk of bias assessment

Risk of bias was assessed by two independent reviewers (KHC and IHC) using the revised Cochrane Risk of Bias tool (RoB 2) 45 across five domains: randomization process, deviations from intended interventions, missing outcome data, outcome measurement, and selective reporting. Each domain was rated as 'low risk,' 'some concerns,' or 'high risk.' The overall study quality was determined by the highest risk rating across all domains. Disagreements were resolved through discussion.

### Data synthesis and analysis

Random-effects meta-analyses were conducted using Comprehensive Meta-Analysis software (vers. 3.0, Biostat, Englewood, NJ, USA). Effect sizes were computed from change scores (mean±standard deviation (SD)) as Hedges' g (standardized mean difference) with 95% confidence intervals (CIs) for continuous outcomes. For dichotomous outcomes, we computed risk ratios with 95% CIs. Statistical significance was evaluated using Z-statistics associated with pooled effect estimates, with a two-tailed *p* value of <0.05 considered significant. Between-study heterogeneity was assessed using I² statistics, with values of 25%, 50%, and 75% respectively considered low, moderate, and high heterogeneity 46. Publication bias was evaluated using funnel plots and Egger's regression test when at least 10 studies were available for analysis 47.

For bone health outcomes, we acknowledge a priori that most included trials employed relatively short intervention durations and assessed total body BMD rather than site-specific measurements at clinically relevant locations such as the lumbar spine or hip 48. Nevertheless, we included these outcomes because they provide preliminary insights into the potential skeletal effects of combined interventions and can inform the design of future longer-term studies with site-specific assessments 18,19,49,50.

Pre-planned subgroup analyses were designed to examine: (1) protein type and dosage (≤20 vs. >20 g/day); (2) exercise modality (resistance alone vs. combined training); (3) intervention duration (≤12 vs. >12 weeks); (4) menopausal status (perimenopausal vs. postmenopausal); and (5) the baseline muscle mass status. However, these analyses were not conducted due to the limited number of included studies, as subgroup analyses require at least 10 studies per subgroup to provide meaningful results according to Cochrane guidelines 51. Sensitivity analyses by excluding studies with a high risk of bias were not performed as no studies were classified as having a high risk of bias in any domain. We assessed the quality of evidence for all meta-analyzed outcomes using the GRADE approach 43.

## Results

### Study selection and characteristics

**Figure 1:**
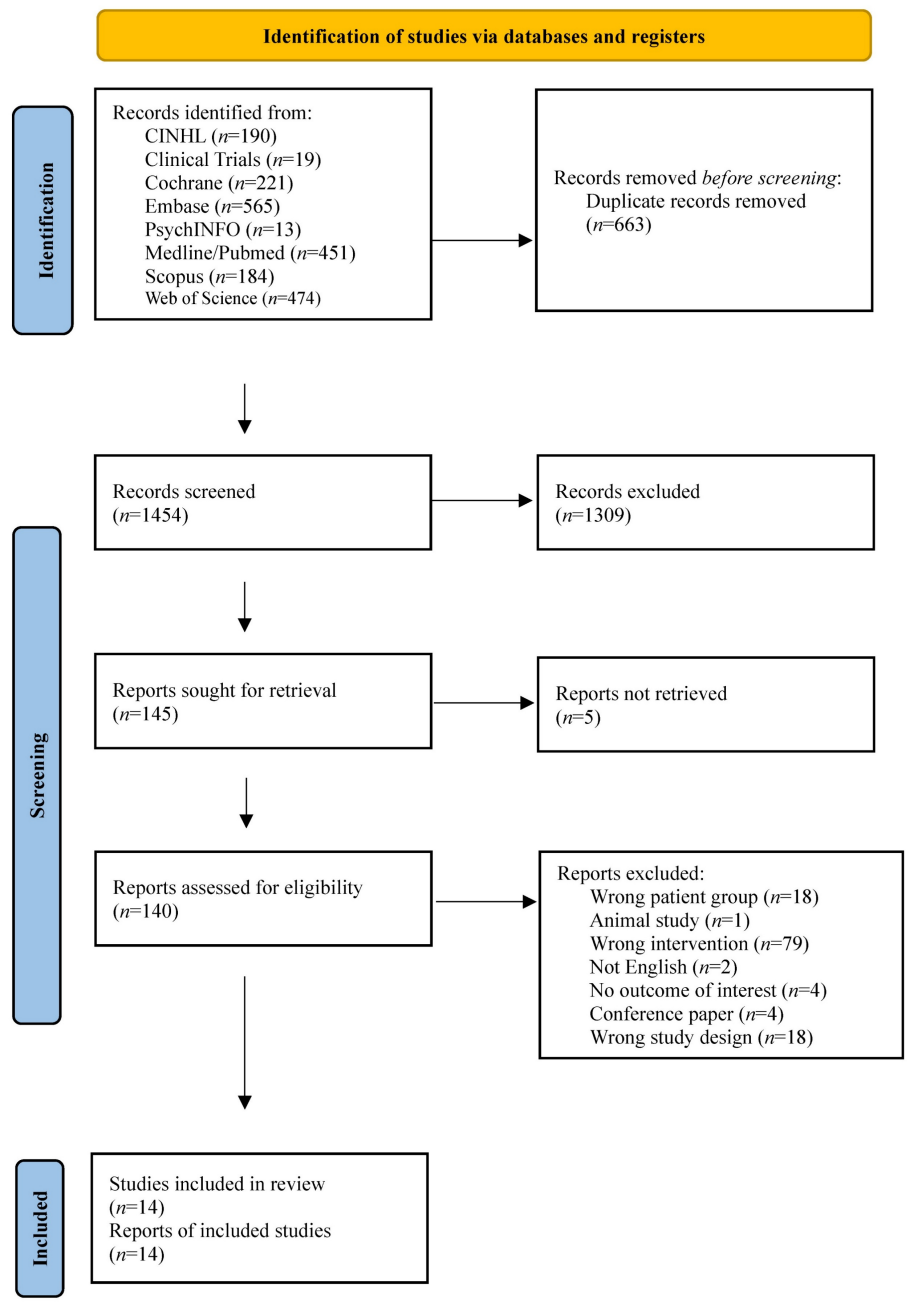
PRISMA flow chart.

Our systematic search yielded 2117 records across eight databases (Supplementary Table S1). After removing 663 duplicates, 1454 records were screened by title and abstract, of which 1309 were excluded. Of the 145 reports sought for retrieval, five could not be obtained. The remaining 140 full-text articles were assessed for eligibility, and 126 were excluded for the following reasons: wrong patient group (*n*=18), animal study (*n*=1), wrong intervention (*n*=79), not English (*n*=2), no outcome of interest (*n*=4), conference paper (*n*=4), and wrong study design (*n*=18). Ultimately, 14 studies met all inclusion criteria (Fig. 1). Included studies were conducted in five countries: the US (five studies 52-56), Brazil (four studies 57-60), Canada (two studies 61,62), Germany (two studies 63,64), and New Zealand (one study 65).

According to Table 1, these studies (published between 2007 and 2025) included 763 participants (386 in the intervention groups and 377 in the comparison groups), with mean ages ranging 32.8-67.1 years, reflecting diverse reproductive stages from premenopausal through postmenopausal years. Two studies exclusively enrolled premenopausal women with mean ages below 42 years 63,65, two studies included women during the perimenopausal transition 52,53, and the remaining studies focused on postmenopausal women with various years since menopause onset 54-62,64. This heterogeneity in reproductive stage was considered acceptable given our research question regarding nutritional supplementation effects across the female lifespan, with particular attention to the menopausal transition as a critical period of accelerated musculoskeletal change 1,2,5-8. Most participants were sedentary or inactive at the baseline (reported in eight of 14 studies 54,55,57,58,61-63,65) and were living in the community (all studies). The body-mass index (BMI) at the baseline ranged 24.0±3.9-35.0±3.4 kg/m² (reported in 13 of 14 studies 52,53,55-65). Among studies reporting years since menopause, the duration ranged 7.6±4.7-9.1±5.2 years 58,59. Use of hormone replacement therapy was explicitly prohibited in most studies. Dropout rates considerably varied from 0% 54,61 to 50% 57.

According to Table 2, studies investigated diverse nutritional supplements rather than exclusively whole-protein supplements. Three studies examined creatine supplementation 60-62, a nitrogen-containing compound synthesized from amino acids that plays a critical role in cellular energy metabolism and has been extensively studied for its effects on muscle mass and strength 33,35,36. Creatine dosages ranged from 0.1 g/kg body weight (BW) to 20 g/day. Other supplements included 9 g of branched-chain amino acids daily 54, 5 g of leucine daily 52,53, 6-10 g of L-citrulline daily 55,56, 15 g of collagen peptides daily 63, 24 g of whey protein daily 65, 25 g of soy protein daily 57,58, combined soy plus milk protein of 37.2 g/day 59, and 2.5 g/kg fat-free mass of a high-protein diet daily 64. This heterogeneity reflects the breadth of nutritional strategies investigated to enhance exercise adaptations in women. Baseline dietary protein intake was reported in eight of 14 studies, and ranged 55.5-83.4 g/day (or 0.8-1.0 g/kg BW/day where reported) 52-54,57-59,61,65. With supplementation, total protein intake would have ranged from approximately 60 to 120 g/day, although actual total intake could only be calculated for studies reporting baseline values.

Exercise protocols predominantly consisted of supervised resistance training performed two or three times per week. One study incorporated both resistance training (three times/week) and walking (six times/week) 61. Two studies used alternative resistance training modalities: whole-body vibration training 55 and slow-velocity, low-intensity resistance training 56. One study combined resistance exercise (twice/week) with interval training (three times/week) 65. Training intensity progressed from 40%-50% to 60%-80% of one-repetition maximum (1RM) in most studies. Intervention durations ranged from 8 54-56 to 96 weeks 61, with a median duration of 16 weeks. Adherence data showed protein supplementation compliance ranging from 56% 61 to 100% 54,58. Exercise adherence ranged from 61% 61 to 100% 58.

### Risk of bias assessment

As shown in Fig. S1, nine of 14 studies (64%) demonstrated a low risk of bias across all domains 52,54,57,58,60-63,65. The remaining five studies (36%) showed some concerns regarding the randomization process, primarily due to insufficient reporting of allocation concealment methods 53,55,56,59,64. All studies demonstrated a low risk of bias for deviations from the intended intervention, missing outcome data, measurement of outcomes, and selection of reported results. The overall methodological quality of the included studies was high, with no study classified as having a high risk of bias in any domain. The concerns in five studies arose from insufficient reporting detail rather than clear evidence of bias; given the low risk of bias across all other domains, we retained all studies in the primary analyses.

### Effects on muscle mass outcomes

**Figure 2:**
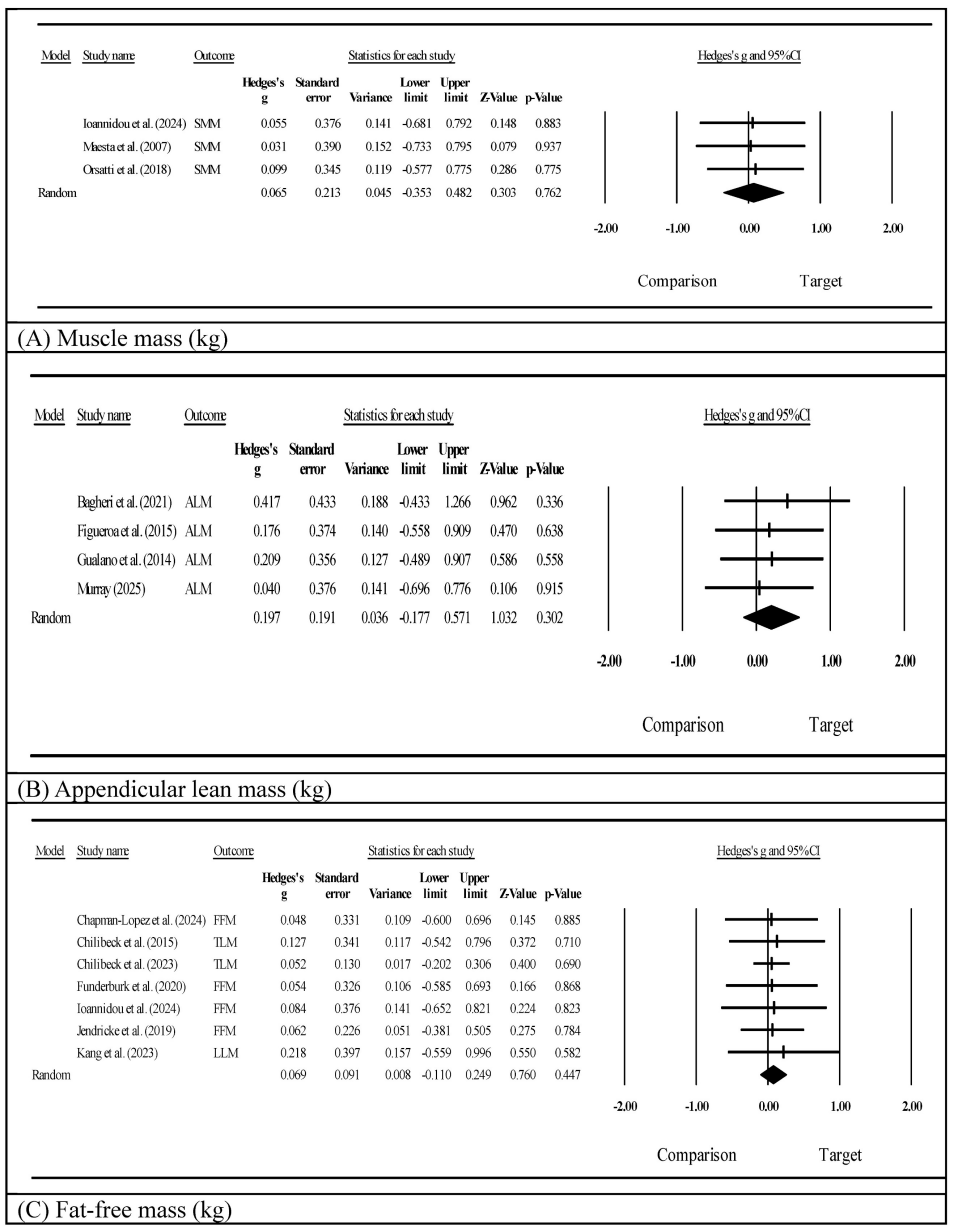
Effects on muscle mass outcomes.

Muscle mass was assessed using either a bioelectrical impedance analysis (BIA; *n*=6) 54,55,57-59,64 or dual-energy x-ray absorptiometry (DXA; *n*=8) 52,53,56,60-63,65 (Supplementary Table S2). Specific muscle mass parameters considerably varied, including appendicular lean mass, fat-free mass, total lean mass, skeletal muscle mass, and the appendicular lean mass index. Because the small number of studies per supplement type precluded formal subgroup meta-analyses 51, we present a narrative summary by supplement category. Among whole-protein studies (k=5) 57-59,64,65, individual studies showed within-group improvements in both the intervention and control groups, but none demonstrated significant between-group differences. Amino acid and derivative studies (k=6) 52-56,63 showed mixed results: Kang et al. 56 (L-citrulline) reported a significant between-group improvement in leg lean mass (*p*<0.05), and Jendricke et al. 63 (collagen peptides) reported improved fat-free mass (d=0.55, *p*<0.05), while the remaining four studies did not reach significance. Creatine studies (k=3) 60-62 also showed mixed findings: Gualano et al. 60 reported a significant between-group improvement in appendicular lean mass (*p*=0.002), whereas Chilibeck et al. 61,62 reported no significance for total lean mass. Overall, no supplement category showed consistently superior effects on muscle mass outcomes. A meta-analysis of muscle mass (skeletal muscle mass, Fig. 2A) showed a statistically non-significant effect favoring protein supplementation combined with exercise (Hedges' g=0.065, 95% CI: -0.353 to 0.482, *p*=0.762, I²=0.000). Appendicular lean mass (Fig. 2B) measured in four studies similarly showed a non-significant positive effect (Hedges' g=0.197, 95% CI: -0.177 to 0.571, *p*=0.302, I²=0.000). Fat-free mass (Fig. 2C) as assessed in seven studies showed a non-significant trend (Hedges' g=0.069, 95% CI: -0.110 to 0.249, *p*=0.447, I²=0.000). All three pooled effect sizes were below the threshold for a small effect (g<0.2), suggesting trivial between-group differences. For the clinical context, individual studies reporting raw values showed within-group changes of approximately 1.2-1.4 kg for muscle mass measures 64, although between-group differences were not statistically significant in any study.

### Muscle strength outcomes

**Figure 3:**
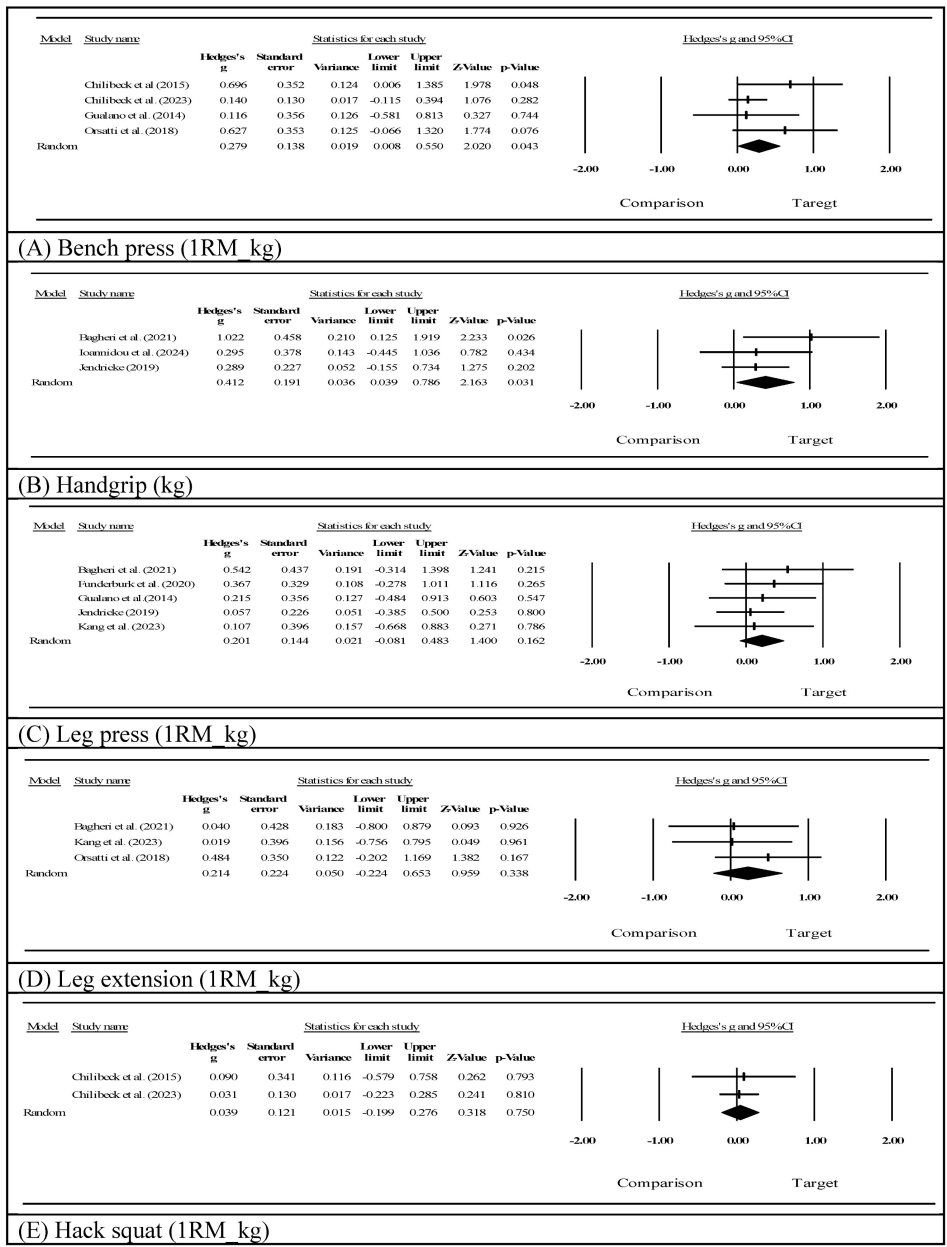
Effects on muscle strength outcomes.

Upper-body strength assessed via bench pressing (Fig. 3A) showed a significant improvement (Hedges' g=0.279, 95% CI: 0.008 to 0.550, *p*=0.043, I²=16.613). Notably, three of the four studies contributing to this outcome involved creatine supplementation 60-62, suggesting that this finding may be largely attributable to creatine's established ergogenic effects on high-intensity resistance exercise 33,35. Handgrip strength (Fig. 3B) demonstrated a statistically significant improvement following the intervention (Hedges' g=0.412, 95% CI: 0.039 to 0.786, *p*=0.031, I²=7.416). In contrast, lower-extremity strength showed favorable but non-significant trends, including leg press 1RM (Fig. 3C, Hedges' g=0.201, 95% CI: -0.081 to 0.483, *p*=0.162, I²=0.000), leg extension strength (Fig. 3D, Hedges' g=0.214, 95% CI: -0.224 to 0.653, *p*=0.338, I²=0.000), and hack squat performance (Fig. 3E, Hedges' g=0.039, 95% CI: -0.199 to 0.276, *p*=0.750, I²=0.000; k=2, very limited evidence).

### Bone health outcomes

Bone health was assessed in five studies 52,60-62,65, with all studies employing DXA for bone mineral measurements. Bone mineral content (Supplementary Fig. S2A) showed a non-significant positive trend favoring the combined intervention (Hedges' g=0.195, 95% CI: -0.281 to 0.671, *p*=0.421, I²=0.000; k=2, very limited evidence). BMD (Fig. S2B) similarly failed to reach statistical significance (Hedges' g=0.087, 95% CI: -0.129 to 0.303, *p*=0.430, I²=0.000). Notably, all six studies assessed total body BMD rather than site-specific measurements (e.g., lumbar spine or femoral neck), and intervention durations ranged 8-96 (median 16) weeks, which may have been insufficient to detect clinically meaningful skeletal changes that typically require 12 months or longer 48,50.

### Body composition measures

There were no significant pooled effects of the intervention on body composition indicators. BW (Fig. S3A) remained stable (Hedges' g=-0.016, 95% CI: -0.272 to 0.239, *p*=0.901, I²=0.000), and no meaningful change in the BMI was observed (Fig. S3B, Hedges' g=-0.033, 95% CI: -0.279 to 0.212, *p*=0.790, I²=0.000). The body fat percentage (Supplementary Fig. S3C) demonstrated a non-significant reduction (Hedges' g=-0.081, 95% CI: -0.303 to 0.141, *p*=0.473, I²=0.000), while visceral adipose tissues (Supplementary Fig. S3D, Hedges' g=-0.111, 95% CI: -0.527 to 0.304, *p*=0.599, I²=0.000), and waist circumference (Supplementary Fig. S3E, Hedges' g=-0.056, 95% CI: -0.601 to 0.488, *p*=0.839, I²=0.000; k=2, very limited evidence) were also unaffected.

### Adverse events

Limited information was provided regarding adverse events across the included studies. No statistically significant differences were observed in adverse event risks between the intervention and control groups (Supplementary Fig. S4, risk ratio (RR)=1.177, 95% CI: 0.510 to 2.717, *p*=0.703; k=2, very limited evidence). Most reported events were mild in nature, including muscle soreness and gastrointestinal discomfort. No serious adverse events were reported in any of the included trials. However, systematic reporting of adverse events was lacking in many of the included trials, and the available data are insufficient to draw definitive conclusions regarding supplement safety.

### GRADE evidence summary

The quality of evidence was assessed using the GRADE approach 43 for all meta-analyzed outcomes and is summarized in Supplementary Table S3. For muscle mass measures, the quality of evidence was rated as low, being downgraded due to imprecision and indirectness. Upper-body strength (bench press and handgrip), BW, BMI, and body fat percentage were rated as low-quality evidence, being downgraded for indirectness and imprecision. Bone health outcomes were rated as very low-quality evidence due to very serious indirectness and serious to very serious imprecision. Overall, the evidence suggested that nutritional supplementation combined with exercise probably makes little or no difference to muscle mass outcomes in women across the menopausal transition.

## Discussion

### Principal findings in context

In this systematic review and meta-analysis of 14 RCTs including 763 women across diverse reproductive stages from premenopausal through postmenopausal years, we evaluated the effects of nutritional supplementation combined with exercise compared to exercise alone or a placebo plus exercise. Our inclusive approach to both participant selection and supplementation type was designed to capture the breadth of evidence regarding nutritional strategies to enhance exercise adaptations in women, with particular attention to the menopausal transition as a period of accelerated musculoskeletal change 1,2,5-8. While pooling mechanistically distinct supplements (whole proteins, amino acids, or creatine) limited causal inferences about specific mechanisms, our approach characterized the current research landscape and provided practical insights into the diverse nutritional strategies studied in this population. Our findings revealed limited additional benefits of nutritional supplementation beyond exercise alone for muscle mass outcomes, with significant improvements observed only for upper-body strength measures. Critically, bone health outcomes assessed in short-duration studies using total body measurements showed non-significant trends (BMD: g=0.087, *p*=0.430; bone mineral content: g=0.195, *p*=0.421) that neither support nor refute potential skeletal benefits; these findings should be considered inconclusive rather than evidence of no effect. Heterogeneity in the reproductive stage, supplementation type, and exercise protocols reflects real-world clinical complexity but also limits our ability to identify optimal intervention strategies for specific populations and precludes definitive mechanistic conclusions about individual supplement types.

The significant improvements in the bench press (g=0.279, *p*=0.043) and handgrip strength (g=0.412, *p*=0.031) without corresponding lower-extremity strength improvements have important functional implications. Lower-extremity strength more directly relates to mobility, balance, and fall prevention--critical factors for fracture risk reduction 11,13,66,67. This selective improvement pattern may reflect distinct fiber type distributions and hormonal sensitivity between the upper- and lower-body musculature in women 68.

### Mechanistic considerations and dose-response relationships

The selective improvement in the bench press may partly be attributable to creatine, as three of four contributing studies included creatine supplementation 60-62. Creatine enhances strength performance through increased phosphocreatine stores and accelerated ATP regeneration 33,35, with effects potentially more pronounced in women whose baseline muscle creatine stores are typically 10%-15% lower than men's 38. The phosphocreatine system provides rapid ATP replenishment during short-duration, high-intensity contractions typical of resistance training, potentially explaining strength gains without corresponding muscle mass increases 36. Dosing protocols varied across the three creatine studies: Chilibeck et al. 61,62 used 0.1-0.14 g/kg BW/day, and Gualano et al. 60 used 20 g/day initially then 5 g maintenance; none followed a loading protocol of 20 g/day for 5-7 days, which expedites muscle creatine saturation, although maintenance-only dosing at 3-5 g/day can achieve comparable saturation over approximately 3-4 weeks 35,37.

The limited efficacy observed for muscle mass outcomes raises important mechanistic questions. While some studies in our review used protein doses (20-37 g/day) similar to those used in older adult studies 29,30, and others used lower doses (5-15 g/day), neither approach showed consistent benefits. Among the eight studies reporting the baseline dietary protein intake, participants consumed 55-83 g/day (0.8 to 1.0 g/kg BW/day) 52-54,57-59,61,65, suggesting that even with supplementation, the total protein intake may have remained below the 1.2-1.6 g/kg BW/day threshold shown to be effective in older adults 30,31,69-70. The unique metabolic environment of an estrogen deficiency may create different protein requirements. An estrogen deficiency induces profound anabolic resistance through multiple pathways: increased inflammatory cytokines (tumor necrosis factor-α and interleukin-6), altered insulin-like growth factor-1 signaling and mitochondrial dysfunction, and increased protein breakdown via the ubiquitin-proteasome pathway 22,23,71. This suggests that women during the menopausal transition may require different supplementation strategies--potentially higher total protein intake levels with leucine enrichment as suggested by Sims et al. 31. Moreover, muscle-bone unit crosstalk through shared regulatory pathways including insulin-like growth factor-1 and myokines 72,73 suggests that standard supplementation doses may be insufficient to enhance exercise-induced anabolic pathways in an estrogen-deficient state 20,21.

### Bone health considerations and measurement limitations

The reliance on total body BMD rather than site-specific measurements at clinically relevant locations (hip, spine, and distal radius) represents a significant limitation of the current evidence 74,75. Total body measurements have lower sensitivity for detecting regional adaptations, particularly at trabecular bone-predominant sites most responsive to interventions 76. Future trials should prioritize site-specific measurements at the lumbar spine and proximal femur, consistent with International Society for Clinical Densitometry recommendations 77.

### Clinical implications and future research directions

Our findings suggest that exercise alone remains the cornerstone intervention for musculoskeletal health in women across reproductive stages 16-19. Current evidence does not support routine nutritional supplementation at the doses studied for enhancing exercise benefits in this population. However, creatine monohydrate may offer targeted benefits for upper-body strength when combined with progressive resistance training, following evidence-based protocols (e.g., a loading phase of 20 g/day for 5-7 days followed by 3-5 g/day maintenance, or 3-5 g/day continuously for 3-4 weeks 35,37). Adequate baseline protein intake also remains important to support exercise adaptations; among the eight studies reporting baseline dietary protein intake, participants consumed below recommended thresholds for older adults (1.2-1.6 g/kg BW/day) 30,69-70, although whether women during the menopausal transition require similar intake levels warrants further investigation.

Exercise programs should emphasize lower-body and multi-joint exercises with progressive overload to maximize functional benefits and fall prevention 12,61,78, given the absence of lower-extremity strength improvements in our analysis. The metabolic context of an estrogen deficiency during and after the menopausal transition may create distinct challenges for supplementation effectiveness 22,24,31, but interventions implemented before menopause may prevent rather than merely treat deterioration 79. The absence of reproductive stage-specific subgroup analyses in our review, due to insufficient study numbers, represents an important knowledge gap.

The lack of detectable bone adaptations in short-duration studies using total body measurements suggests that adequate baseline nutrition, optimal exercise programming, and a sufficient intervention duration all require consideration for bone health optimization during this critical transition 80. Several priority areas for future investigation emerged from our findings: (1) adequately powered trials with intervention durations of at least 12 months and site-specific bone density assessments, incorporating bone turnover markers to evaluate early biochemical responses 18,19,48,49,81; (2) dose-response studies examining whether higher protein intakes or specific amino acid compositions can overcome estrogen deficiency-related anabolic resistance 22,24,31,82; (3) comparative effectiveness trials directly comparing supplement types, as our findings suggested that creatine may offer particular advantages for upper-body strength 33,35-38,60-62; (4) functional outcomes including gait speed, balance, fall incidence, and fracture occurrence 11,13,66,83; and (5) standardized adverse event monitoring and reporting protocols, as the current evidence base lacks systematic safety data to inform clinical recommendations.

### Strengths and limitations

This systematic review represents a comprehensive synthesis of evidence specifically examining combined nutritional and exercise interventions in women across the menopausal transition, addressing a critical knowledge gap 84. Our pragmatic, inclusive approach to supplement selection provides a broad overview of nutritional strategies studied in this population. The inclusion of diverse protein types and control conditions enhanced the generalizability of the findings. The generally high methodological quality of the included studies, with 64% demonstrating a low risk of bias across all domains, strengthens confidence in our results.

Several important methodological limitations warrant acknowledgment. First, our inclusive approach to participant selection, encompassing women from premenopausal through postmenopausal stages, created heterogeneity that may have obscured stage-specific effects. While this breadth provided insights across the female lifespan 85, it limited our ability to draw definitive conclusions specifically about the menopausal transition. The two studies including exclusively premenopausal women 63,65 represent a distinct metabolic context compared to studies in women experiencing an estrogen deficiency 22,24, yet our decision to retain those studies reflects the clinical reality that musculoskeletal health optimization is relevant across all reproductive stages 86, and preventive interventions before menopause may prove more effective than interventions after deterioration is established 87. Second, our pooling of mechanistically distinct nutritional supplements—whole proteins, isolated amino acids, and creatine—represents a key methodological consideration. The mechanisms of actions of these compounds substantially differ: whole proteins provide complete amino acid profiles for muscle protein synthesis, isolated amino acids function as specific anabolic signals, and creatine primarily affects cellular energy metabolism through phosphocreatine stores rather than serving as a protein building block 33,35-36. Pooling these interventions limited the mechanistic interpretability of our effect estimates and prevented isolation of supplement-specific effects. We adopted this approach because: (a) these supplements are frequently studied together in exercise nutrition research as complementary strategies 25,33,35-38,40; (b) the small number of studies per supplement type (three for creatine, and two or three for other types) precluded adequately powered separate analyses according to Cochrane guidelines requiring ≥10 studies per subgroup 51; and (c) our objective was to characterize the breadth of nutritional approaches studied in this population rather than to establish causal mechanisms for individual supplements. This heterogeneity is reflected in our GRADE ratings, which were downgraded for indirectness. Readers should interpret our pooled estimates as descriptive summaries of the current literature rather than as precise estimates of the effects of specific supplements. Future research should conduct adequately powered head-to-head comparisons of specific supplement types to provide clearer mechanistic and clinical guidance. Third, bone health outcomes were predominantly assessed using total body measurements over short intervention durations, limiting our ability to evaluate the fracture prevention potential. The median study duration of 16 weeks was insufficient for detecting meaningful changes in BMD, which typically requires 12 months or longer in postmenopausal women 18,19,64,88. Furthermore, total body measurements might not reflect regional adaptations at weight-bearing sites most relevant to osteoporotic fracture risk 89. The absence of site-specific measurements of the lumbar spine, femoral neck, or radius represents a significant limitation 77. Additionally, no included studies reported bone turnover markers, which could have provided insights into early skeletal responses over shorter time frames 90. The small number of included studies (*n*=14) precluded planned subgroup analyses that could identify responder characteristics 51. Only eight of 14 studies reported the baseline dietary protein intake 52-54,57-59,61,65, limiting our ability to determine whether the total protein intake reached recommended thresholds. Substantial heterogeneity in intervention protocols and the relatively short median duration of 16 weeks may have been insufficient for detecting changes in bone tissues, which typically requires 6-12 months for measurable adaptations 91. The limited assessment of functional outcomes directly related to fall risks 12,92 and the absence of fracture data limited the clinical applicability of findings for osteoporosis prevention. The exclusion of gray literature may have introduced publication bias.

## Conclusions

Results of this systematic review demonstrate that nutritional supplementation does not provide consistent additive benefits for muscle mass when combined with exercise in women across reproductive stages. While selective improvements in upper-body strength were observed, particularly in studies incorporating creatine supplementation, lower-extremity strength and muscle mass outcomes showed no significant enhancements. Bone health outcomes assessed in short-duration studies using total body measurements showed non-significant trends, with study limitations precluding definitive conclusions regarding the fracture prevention potential. For clinical practice, exercise alone currently provides the most reliable benefits for musculoskeletal health across women's reproductive stages. Current evidence does not support routine nutritional supplementation at the doses studied for enhancing muscle mass or bone health benefits of exercise. Adequate baseline protein intake remains important to support exercise adaptations, although optimal intake levels for women during the menopausal transition remain to be determined.

## Supplementary Material

Supplementary figures and tables.

## Figures and Tables

**Table 1:** Study characteristics of the included articles

First author (year) country	Health status	Intervention group						Comparison group					
		*N*/*n* ^a^	Age, years (mean±SD)	BMI. kg/m^2^ (SD)	Years since menopause (mean±SD)	Hormone replacement therapy use (%)	Dropout rates (%)	*N*/*n* ^a^	Age, years (mean±SD)	BMI, kg/m^2^ (SD)	Years since menopause (mean±SD)	Hormone replacement therapy use (%)	Dropout rates (%)
Bagheri (2021) 54 USA	Postmenopausal (≥1 year without menstruation), sedentary, healthy	10/10	NR^*^	NR	NR	No	0	10/10	NR^*^	NR	NR	No	0
Chapman-Lopez (2024) 52 USA	Perimenopausal/postmenopausal (self-identification)	23/17	55.76±7.43	27.7 (4.7)	NR	NR	26	23/18	53.50±6.20	28.6 (5.6)	NR	NR	22
Chilibeck (2015) 62 Canada	Postmenopausal (a questionnaire regarding menstrual period + FSH/ luteinizing hormone if <2 years)	23/15	57.0±4.0^‡^	26.6 (4.2)	NR	No	43	24/18	57.0±7.0^‡^	27.5 (6.0)	NR	No	26
Chilibeck (2023) 61 Canada	Postmenopausal (a questionnaire regarding menstrual period + FSH/ luteinizing hormone if <2 years), low & moderate risk of osteoporosis	120/120	59.0±5.6	27.3 (5.9)	NR	No	0	117/117	59.0±5.7	27.0 (5.7)	NR	No	2.5
Figueroa (2015) 55 USA	Postmenopausal (≥1 year without menstruation), BMI ≥ 30 kg/m^2^, prehypertension or stage-1 HTN, nonsmokers, sedentary	13/13	58.0±3.6	33.8 (4.0)	NR	No	NR	14/14	58.0±3.7	35.0 (3.4)	NR	No	NR
Funderburk (2020) 53 USA	Perimenopausal/postmenopausal (self-identification), healthy	23/18	53.5±6.2	NR	NR^⁋^	NR	22	23/18	55.6±7.2	NR	NR^⁋^	NR	22
Gualano (2014) 60 Brazil	Postmenopausal, osteopenia or osteoporosis	19/15	67.1±5.6	28.0 (2.1)	NR	NR	21	19/15	63.6±3.6	28.2 (3.6)	NR	NR	21
Ioannidou (2024) 64 Germany	Postmenopausal (last period >2 years ago + low E2 and P levels confirmed by saliva test), healthy	NR/15	57.0±6.1	25.8 (3.8)	NR	0	NR^‡^	NR/12	57.6±5.1	24.5 (3.4)	NR	0	NR^‡^
Jendricke (2019) 63 Germany	Premenopausal (a comprehensive anamnesis)	45/40	38.3±8.7	26.4 (3.8)	NA	NA	11	45/37	41.6±6.9	26.5 (3.4)	NR	NA	18
Kang (2023) 56 USA	Postmenopausal (≥1 year without menstruation), sedentary, HTN	14/13	62.0±2.0	29.6 (4.0)	NR	29	7	14/11	63.0±1.0	29.2 (5.6)	NR	21	21
Maesta (2007) 59 Brazil	Postmenopausal (>1 year amenorrhea+FSH >40 mIU/mL)	14/14	57.6±6.7	27.8 (4.0)	8.1±5.3	No	NR^†^	11/11	60.7±7.1	27.7 (4.4)	7.6±4.7	No	NR^†^
Murray (2025) 65 New Zealand	Premenopausal, healthy, nonsmokers, BMI >18.5 <30 kg/m^2^	16/15	34.2±9.1	24.0 (3.9)	NA	NA	6	15/12	32.8±9.7	27.1 (3.1)	NA	NA	20
Orsatti (2018) 58 Brazil	Postmenopausal (>1 year amenorrhea+FSH >40 mIU/mL), healthy, sedentary	21/16	56.8±6.6	27.5 (4.0)	7.6±5.2	No	24	20/16	58.8±8.9	27.3 (4.1)	9.1±5.2	No	20
Trevisan (2010) 57 Brazil	Postmenopausal (>1 year amenorrhea+FSH >40 mIU/mL), sedentary	30/15	58.0±7.0	28.0 (4.0)	NR	No	50	30/15	57.0±9.0	28.0 (5.0)	NR	No	50

^a^ Sample sizes given as *N* (recruited)/*n* (included in analysis).^‡^ Sample age at recruitment. BMI, body-mass index; E2, estradiol; P, progesterone; NR, not reported; HTN, hypertension; FSH, follicle-stimulating hormone; NA, not available. NR^*^ Not reported stratified for intervention and control groups, total sample age 56±3.7 years. NR^⁋^ Only reported postmenopausal women were with a mean duration of 7 years since the onset of menopause. NR^†^ Not reported stratified for intervention and control groups, total dropout rate 23%. NR^‡^ Not reported stratified for intervention and control groups, total dropout rate 13%. Note: Studies included women across diverse reproductive stages from premenopausal through postmenopausal years. Menopausal status definitions were as reported in original studies.

**Table 2:** Intervention characteristics of the included articles (nutritional supplements and exercise protocols)

First author	Intervention group									Comparison group	
	Intervention	Exercise	Dose per serving (g)	Total dose (g/day)	Frequency	Timing	Duration (weeks)	Adherence (%)	Baseline protein intake (g/day)	Intervention	Baseline protein intake (g/day)
Bagheri 54	BCAA+supervised resistance exercise	Progressed from 3×12 @60%-65% 1RM to 4×10 @70%-75%.	9 (4.5 g leucine+2.25 g isoleucine+2.25 g valine)	9	3 times/week	Half 30 min before & half within 30 min following exercise	8	100 (questionnaire+container return)	55.5±9.0	PL+supervised resistance exercise Both: 100	57.2±11
Chapman-Lopez 52	Leucine+supervised resistance exercise	Weeks 1-3: 1-2 sets of upper- & lower-body machines. Weeks 4-10: 3 sets × 10 reps. Progressive overload. Equipment: machines.	5	5	3 times/week	Immediately after exercise	10	Leucine: 99.1±1.4 Exercise: 97.6±3.8 (attendance+pill count)	83.4±29.3	PL(apple pectin 5 g/day)+supervised resistance exercise PL: 96.3±3.2 Exercise: 95.8±3.7	68.3±40.9
Chilibeck 62	Creatine+supervised resistance exercise	3 sets × 10 reps to fatigue. Intensity ≈80% 1RM for hack squat and bench press; ≈10 RM for other exercises. Progressive overload applied individually. ~15 exercises/session (e.g., hack squat, multihip machine, curls, presses, lat pulldown, dumbbells). Equipment: free weights, machines.	NA	0.1 g/kg	Creatine: 7 days/week Exercise: 3 times/week	Immediately before (0.05 g/kg) & after (0.05 g/kg) exercise With two meals (0.05 g/kg) on non-training days	48	Creatine: 79 Exercise: 75 (117/156 sessions) (attendance logs, pill count, diaries)	NR	PL (maltodextrin)+supervised resistance exercise PL: 78 Exercise: 77 (120/156 sessions)	NR
Chilibeck 61	Creatine+supervised resistance/walking exercise	Resistance: 2 sets × 8 reps to muscular fatigue at ≥80% 1RM or 8 RM. Progressive overload by 2-5 kg when able to complete 2 × 8. ~12 exercises/session (hack squat, hip machines, bench press, etc.). Equipment: plate-loaded and weight-stack machines. Walking: 20-30 min/session at 70% HR max.	0.14 g/kg creatine+0.14 g/kg maltodextrin	0.14 g/kg	Creatine: 7 days/week Exercise: 3 times/week resistance; 6 times/week walking (3 supervised+3 unsupervised)	Half 5 min post resistance & half with a meal 1/3 with each meal on non-training days	96	Creatine: 56 Resistance: 61 Walking: 65 (pill count, logs)	67.0±22.0	PL (maltodextrin 0.28 g/kg/day)+supervised resistance/walking exercise PL: 60 Resistance: 61 Walking: 60	64.0±23.0
Figueroa 55	L-citrulline+WBVT	4 static + 4 dynamic leg exercises Equipment: vibration platform (pro5 AIRdaptive). Intensity: vibration 25-40 Hz, amplitude 1-2 mm. Sets × Reps: 1-5 sets, 30-60 s per set; dynamic exercises at 3 s eccentric / 2 s concentric pace. Progressive overload: increase vibration intensity, duration, # sets, total session time (11-60 min); decrease rest (60→30 s).	0.75	6	L-citrulline: 7 days/week WBVT: 3 times/week	Half before breakfast & half before sleep	8	L-citrulline: 96.4±3.6 WBVT: 99.7±1.1 (capsule return count, logs)	NR	PL (maltodextrin)+WBVT PL: 95.8±3.0 WBVT: 99.7±1.1	NR
Funderburk 53	Leucine+supervised resistance exercise	Weeks 1-2: 2×10 reps at 80% 1RM; Weeks 3-10: 3×10 reps. Load adjusted to reach volitional exhaustion in final set. 6 resistance machine exercises: leg press, leg extension, seated leg curl, shoulder press, bench press, seated row. 60 s rest between sets.	0.5	5	Leucine: 7 days/week Exercise: 3 times/week	Immediately after exercise With first meal on non-training days	10	Leucine: 99.1±1.4 Exercise: 97.6±3.8 (capsule count, verbal check every 2 weeks, attendance logs)	83.4±29.3	PL (apple pectin 5 g/day)+supervised resistance exercises PL: 96.3±3.1 Exercise: 95.8±3.7	68.2±39.6
Gualano 60	Creatine+supervised resistance exercise	Week 1: 2 sets of 15-20 RM; Weeks 2-24: 3 sets of 8-12 RM. Load progressed when >12 reps were achieved in a set. Exercises: leg press, leg extension, squat, seated row, bench press, lat pulldown, sit-ups.	5	20	Creatine: 7 days/week Exercise: 2 times/week	Breakfast, lunch, dinner & before bed time on training days Lunch on non-training days	24	Creatine: NR Exercise: 84.4±8.0	56.0±13.0	PL (dextrose 20 g/day)+supervised resistance exercise PL: NR Exercise: 83.9±6.1	58.0±18.0
Ioannidou 64	Protein+supervised resistance exercise	Each session: 6 sets, reps from 10→3, RIR from 8→1. Rest: 60-180 s based on RIR. Includes peak (1RM test) and deload weeks (↓30% load). Tempo: 1 s concentric / 1 s hold / 1 s eccentric. Exercises: box back squat, deadlift, lat pulldown, lateral dumbbell raises, single arm lateral walking carries.	Daily: Whey & protein-rich cheese 2.5 g/kg FFM/day Post-training: 30 g whey+40 g maltodextrin	Daily: 2.5 g/kg FFM/day Post-training: 30	Protein: 7 days/week Post-training: 3 days/week Exercise: 3 times/week	Daily: NR Post-training	12	NR	NR	Supervised resistance exercise Exercise: NR	NR
Jendricke 63	Collagen peptides+ Supervised resistance exercise	Exercises: leg press, back trainer, lat pull-down, sit-ups, chest press. Sets: 3 per exercise. Reps progressed: Weeks 1&2: 15 reps; Weeks 3&4: 12 reps; Weeks 5-8: 10 reps; Weeks 9-12: 8 reps. Load adjusted to maintain rep targets with proper form.	NA	15	Collagen: 7 days/week Exercise: 3 times/week	60 min after exercise Same time on non-training days	12	NR (missing >6 sessions were excluded)	NR	PL (silicon dioxide)+ supervised resistance exercise	NR
Kang 56	L-citrulline+ SVLIRT	Exercises: leg press, leg extension, leg curl, calf raise. Intensity: Weeks 1&2 at 40% 1RM; Weeks 3&4 at 50% 1RM. Tempo: 3 s concentric + 3 s eccentric using metronome. Volume: 3 sets × 15 reps; rest: 1-3 min.	0.77	10	L-citrulline: 7 days/week SVLIRT: 3 times/week	4.62 g in morning & 5.39 g at night	L-citrulline: 8 SVLIRT: 4	L-citrulline: 96±6^†^ SVLIRT: 95±7^†^ (capsule return, biweekly phone check, Attendance logs)	NR	PL (maltodextrin 10 g/day)+ SVLIRT PL: 96±6^†^ SVLIRT: 95±7^†^	NR
Maesta 59	Soy+milk protein+supervised resistance exercise	Week 1: 1 set of 15 reps at 40%-50% 1RM; progressed to 3 sets of 8-12 reps at 60%-80% 1RM. Monthly load adjustments. Full-body workout (40-50 min): 2 exercises per major muscle group (chest, back, thighs), 1 per minor group (biceps, triceps). Exercises included: leg press, leg extension, peck deck, bench press, seated row, lat pulldown, triceps pulley, biceps curl, plus abs (3×30) and calves (3×20 BW).	37.2	Soy: 25 Milk: 12.2	Soy+milk: NR Exercise: 3 times/week	NR	16	NR	NR	PL (maltodextrine 25 g/day) +supervised resistance exercise	NR
Murray 65	Whey protein+supervised resistance exercise/interval training	Resistance: upper body dumbbell training. Exercises: chest press, single-arm row, shoulder press; other upper-body & abs rotated (not recorded). Load progressed via “rule of two”; adjusted per 3RM. Interval: cycling intervals. Sprint = RPE 10, rest = RPE 2. Intervals progressed from 6 to 9.	24	24	Whey protein 5 days/week Resistance: 2 times/week (48 h apart) Interval: 3 times/week	After exercise	Whey protein 12 Resistance 9 Interval 12	Whey protein NR Resistance 89±7 Interval 82±13 (attendance logs)	70±20 g/day	PL (Milo powder)+supervised resistance exercise/interval training PL: NR Resistance: 91±7 Interval: 89±8	79±12 g/day
Orsatti 58	Soy protein+ supervised resistance exercise	Week 1 - 1×15 reps at 40%-50% 1RM; Week 4 - 3×8-12 reps at 60%-80% 1RM. Monthly load adjustments (0%-5%) to maintain 8-12 RM range. Exercises: leg press, leg extension, leg curl, fly, bench press, seated row, lat pulldown, triceps pulley, biceps curl, abs (3×30), calves (3×20 BW).	25	25	Soy protein: 7 days/week Exercise: 3 times/week	After exercise Same time on non-training days	16	Soy: NR Exercise: 100	1.0±0.3 g/kg	PL (maltodextrin 25 g/day)+ supervised resistance exercise PL: NR Exercise: 100	0.8±0.3 g/kg
Trevisan 57	Soy protein+ supervised resistance exercise	First 4 weeks aimed at physical aptitude equalization. Warm-up: 10 unloaded reps before each exercise; post-session: 15 s static stretch per muscle group. 10 exercises: leg press, leg extension, leg curl, bench press, pec-deck fly, seated row, lat pulldown, triceps pulley, barbell curls, abdominal crunches (3×30 reps). Progressed to 3 sets of 8-12 reps at 60%-80% 1RM with periodic adjustments. 1-2 min rest between sets; 1-2 s pause between reps. All sessions used appropriate machines	25	25	Soy protein: 7 days/week Exercise: 3 times/week	NR	16	NR	60.0±21.0	PL (maltodextrin)+supervised resistance exercise	59.0±39.0

BCAA, branched-chain amino acid; FFM, fat-free mass; RM, repetition maximum; 1RM, one repetition maximum; PL, placebo; NA, not applicable; NR, not reported; RIR, repetitions in reserve. RPE, rating of perceived exertion; WBVT, whole-body vibration exercise training; SVLIRT, slow-velocity low-intensity resistance training. ^†^ Mean of all participants.

## Data Availability

All data supporting the findings of this study are included within the study. Additional information can be obtained from the corresponding authors upon reasonable request.

## Abbreviations

ALM: appendicular lean mass
BIA: bioelectrical impedance analysis
BMC: bone mineral content
BMD: bone mineral density
BMI: body mass index
BW: body weight
CI: confidence interval
DXA: dual-energy x-ray absorptiometry
FFM: fat-free mass
GRADE: Grading of Recommendations Assessment, Development and Evaluation
PRISMA: Preferred Reporting Items for Systematic Reviews and Meta-Analyses
PROSPERO: International Prospective Register of Systematic Reviews
RCT: randomized controlled trial
RoB: risk of bias
RR: risk ratio
SD: standard deviation
SMD: standardized mean difference
