# Supplementary material for: Nutritional Supplementation Combined with Exercise for Musculoskeletal Health in Women: A Systematic Review and Meta-Analysis Evaluating Proteins, Amino Acids, and Creatine across Reproductive Stages
Source: Int J Med Sci. 2026 Apr 16;23(6):1933–51. doi: 10.7150/ijms.130435 (PMC13181379; doi:10.7150/ijms.130435)
Supplement: Supplementary file 1 — Supplementary figures and tables. [file ijmsv23p1933s1.pdf]

Supplementary Table S1. Complete search strategies for all databases

#	Database	Search Strategy	Results
1	CINAHL	(MH "Menopause+" OR TI menopaus* OR AB menopaus* OR TI postmenopaus* OR AB postmenopaus* OR TI perimenopaus* OR AB perimenopaus*) AND (MH "Dietary Proteins+" OR MH "Amino Acids+" OR MH "Dietary Supplements+" OR TI protein* OR AB protein* OR TI "amino acid*" OR AB "amino acid*" OR TI supplement* OR AB supplement*) AND (MH "Exercise+" OR MH "Resistance Training+" OR MH "Physical Activity+" OR TI exercise OR AB exercise OR TI "resistance training" OR AB "resistance training" OR TI "physical activity" OR AB "physical activity") AND (MH "Muscle Mass" OR MH "Lean Body Mass" OR MH "Sarcopenia" OR MH "Bone Density+" OR TI "muscle mass" OR AB "muscle mass" OR TI "lean mass" OR AB "lean mass" OR TI sarcopenia OR AB sarcopenia OR TI "bone density" OR AB "bone density")	190
2	ClinicalTrials.gov	Condition/disease: (menopause OR postmenopaus OR perimenopaus*) Other terms: (protein OR "amino acid" OR supplement) AND (exercise OR "resistance training" OR "physical activity") AND ("muscle mass" OR "lean mass" OR sarcopenia OR "bone density")	19
3	Cochrane CENTRAL	("menopause" OR "postmenopause" OR "perimenopause"):ti,ab,kw AND ("protein*" OR "amino acid*" OR "supplement*"):ti,ab,kw AND ("exercise" OR "resistance training" OR "physical activity"):ti,ab,kw AND ("muscle mass" OR "lean mass" OR "sarcopenia" OR "bone density"):ti,ab,kw	221
4	Embase	('menopause'/exp OR 'postmenopause'/exp OR perimenopaus*:ti,ab) AND ('protein'/exp OR 'amino acid'/exp OR supplement*:ti,ab) AND ('exercise'/exp OR 'resistance training'/exp OR 'physical activity'/exp) AND ('muscle mass'/exp OR 'lean body mass'/exp OR 'sarcopenia'/exp OR 'bone density'/exp)	565
5	PsycINFO	(menopause OR postmenopaus OR perimenopaus*) AND (protein* OR amino acid* OR supplement*) AND (exercise OR resistance training OR physical activity) AND (muscle mass OR lean mass OR sarcopenia OR bone density)	13
6	MEDLINE/PubMed	(menopause OR postmenopaus OR perimenopaus*) AND (protein* OR amino acid* OR supplement*) AND (exercise OR resistance training OR physical activity) AND (muscle mass OR lean mass OR sarcopenia OR bone density)	451
7	Scopus	TITLE-ABS("menopause" OR "postmenopaus*" OR "perimenopaus*") AND TITLE-ABS("protein*" OR "amino acid*" OR "supplement*") AND TITLE-ABS("exercise" OR "resistance training" OR "physical activity") AND TITLE-ABS("muscle mass" OR "lean mass" OR "sarcopenia" OR "bone density")	184
8	Web of Science	TS=(menopause OR postmenopaus* OR perimenopaus*) AND TS=(protein* OR "amino acid*" OR supplement*) AND TS=(exercise OR "resistance training" OR "physical activity") AND TS=("muscle mass" OR "lean mass" OR sarcopenia OR "bone density")	474

All searches were conducted from database inception through July 31, 2025. Search strategies were adapted to each database’s controlled vocabulary and syntax. No language or date filters were applied at the search level.

Supplementary Table S2. Muscle mass measures and the effect of the intervention group compared to the control group

First author (year)	Method	Measure, units	Assessment time/Fasting duration	Intervention group			Comparison group			Intervention effects	
				Pre	Post	Change [a]	Pre	Post	Change [a]	MD [b]	*p*
Bagheri [54]	BIA	ALM, kg	NR/NR (bladder voided)	NR	NR	1.9<br>3.1[c]	NR	NR	1.4<br>2.8[c]	NR	NR
Chapman-Lopez [52]	DXA	FFM, kg	NRNR	43.1±7.0	43.8±6.5	NR	43.0±5.4	43.4±5.5	NR	NR	NS
Chilibeck [62]	DXA	TLM, kg	NR/NR	NR	NR	-1.0[d]<br>(-2.1, 0.8)	NR	NR	-1.3[d]<br>(-2.3, -0.3)	NR	NR
Chilibeck [61]	DXA	TLM, kg	NR/NR	41.4±6.2	43.5±6.3	NR	40.3±5.3	42.1±5.4	NR	NR	NS
Figueroa [55]	DXA	ALMI, kg/m^2	Morning (±1 h)/overnight fast	7.6 ± 0.3[e]	7.9 ± 0.3[e]	NR	8.1±0.3[e]	8.2 ± 0.3[e]	NR	NR	NR
Funderburk [53]	DXA	FFM, kg	NR/NR	43.1±0.7	43.8±0.7	NR	43.0±0.5	43.5±5.3	NR	NR	NS
Gualano [60]	DXA	ALM, kg	NR/NR	16.8±2.3	17.1±2.0	1.31[f]	17.4±1.5	17.3±1.5	-0.2[f]	NR	0.002
Ioannidou [64]	BIA	SMM, kg<br>FFM, kg	NR/NR	19.8±3.3	21.2±3.5	1.4±0.9	20.6±3.4	21.8±3.8	1.2±1.3	NR	NS
Jendricke [63]	BIA	FFM, %	Morning (07:00–11:00)/NR	62.6±6.0	64.4±6.2	1.8±1.6<br>(1.0±0.9 kg)	63.8±6.0	64.7±6.0	0.9±1.6<br>(0.4±0.9 kg)	0.55[c]	<0.05
Kang [56]	DXA	LLM, kg	NR/NR	13.5±0.6[e]	14.0 ± 0.7[e]	NR	12.4±0.5[e]	12.4±0.5[e]	NR	NR	<0.05
Maesta [59]	BIA	SMM, kg	NR/≥4 h fast; no caffeine/alcohol 12 h prior; no exercise 24 h prior; 2 L water day before; bladder emptied	19.7±3.3	21.0±3.6	1.3±0.9	17.8±2.5	19.2±2.8	1.3±1.2	NR	NR
Murray [65]	DXA	ALM, kg	NR/NR	18.1±3.1	18.7±2.8	0.6±2.3	19.8±1.5	20.5±1.5	0.7±1.2	-0.1±0.7	NS
Orsatti [58]	BIA	SMM, kg	NR/NR	19.5±3.1	20.7±3.5	1.2[d]<br>(0.7, 1.7)	18.0±2.4	19.5±2.7	1.5[d]<br>(1.0, 1.9)	NR	NS
Trevisan [57]	BIA	SMI, %	NR/NR (bladder voided)	28.0±3.0	NR	NR	28.0±4.0	NR	NR	NR	NR

Muscle mass measures are presented as the mean±standard deviation unless otherwise stated.

[a] Change reported as the muscle mass post-intervention minus pre-intervention. [b] Mean difference defined as the mean change of muscle mass measure in the intervention group minus the mean change of muscle mass measure in the control group. [c] Cohen's d effect size. [d] Mean (95% confidence interval). [e] Mean (standard error). [f] Percentage.

BIA, bioelectrical impedance analysis; ALM, appendicular lean mass; NR, not reported; DXA, dual-energy x-ray absorptiometry; FFM, fat-free mass; NS, not significant; TLM, total lean mass; ALMI, appendicular lean mass index (calculated as ALM/height in m^2); LLM, leg lean mass; SMM, skeletal muscle mass; SMI, skeletal mass index (calculated as SMM/weight in kg).

Supplementary Table S3. GRADE evidence profile

Outcome	No. of studies (no. of participants)	Risk of bias	Inconsistency	Indirectness	Imprecision	Publication bias	Effect estimate (95% CI)	Quality of evidence	Comments
Muscle mass	3 (84)	Not serious[1]	Not serious[2]	Serious[3]	Serious[4]	Undetected[5]	0.065 (-0.353 to 0.482)	⊕⊕◯◯ LOW	Downgraded 2 levels: -1 for indirectness, -1 for imprecision
Appendicular lean mass	4 (114)	Not serious[1]	Not serious[2]	Serious[3]	Serious[4]	Undetected[5]	0.197 (-0.177 to 0.571)	⊕⊕◯◯ LOW	Downgraded 2 levels: -1 for indirectness, -1 for imprecision
Fat-free mass	7 (469)	Not serious[1]	Not serious[2]	Serious[3]	Serious[4]	Undetected[5]	0.069 (-0.110 to 0.249)	⊕⊕◯◯ LOW	Downgraded 2 levels: -1 for indirectness, -1 for imprecision
Bench press	4 (332)	Not serious[1]	Not serious[2]	Serious[3]	Serious[6]	Undetected[5]	0.279 (0.008 to 0.550)	⊕⊕◯◯ LOW	Downgraded 2 levels: -1 for indirectness, -1 for imprecision
Handgrip	3 (134)	Not serious[1]	Not serious[2]	Serious[3]	Serious[6]	Undetected[5]	0.412 (0.039 to 0.786)	⊕⊕◯◯ LOW	Downgraded 2 levels: -1 for indirectness, -1 for imprecision
Leg press	5 (187)	Not serious[1]	Not serious[2]	Serious[3]	Serious[4]	Undetected[5]	0.201 (-0.081 to 0.483)	⊕⊕◯◯ LOW	Downgraded 2 levels: -1 for indirectness, -1 for imprecision
Leg extension	3 (76)	Not serious[1]	Not serious[2]	Serious[3]	Serious[4]	Undetected[5]	0.214 (-0.224 to 0.653)	⊕⊕◯◯ LOW	Downgraded 2 levels: -1 for indirectness, -1 for imprecision
Hack squat	2 (270)	Not serious[1]	Not serious[2]	Serious[3]	Serious[4]	Undetected[5]	0.039 (-0.199 to 0.276)	⊕⊕◯◯ LOW	Downgraded 2 levels: -1 for indirectness, -1 for imprecision
Bone mineral content	2 (65)	Not serious[1]	Not serious[2]	Very serious[7]	Very serious[8]	Undetected[5]	0.195 (-0.281 to 0.671)	⊕◯◯◯ VERY LOW	Downgraded 4 levels: -2 for very serious indirectness, -2 for very serious imprecision
Bone mineral density	4 (326)	Not serious[1]	Not serious[2]	Very serious[7]	Serious[4]	Undetected[5]	0.087 (-0.129 to 0.303)	⊕◯◯◯ VERY LOW	Downgraded 3 levels: -2 for very serious indirectness, -1 for imprecision
Body weight	6 (226)	Not serious[1]	Not serious[2]	Serious[3]	Serious[6]	Undetected[5]	-0.016 (-0.272 to 0.239)	⊕⊕◯◯ LOW	Downgraded 2 levels: -1 for indirectness, -1 for imprecision
Body-mass index	7 (244)	Not serious[1]	Not serious[2]	Serious[3]	Serious[6]	Undetected[5]	-0.033 (-0.279 to 0.212)	⊕⊕◯◯ LOW	Downgraded 2 levels: -1 for

									indirectness, -1 for imprecision
Body fat	9 (298)	Not serious[1]	Not serious[2]	Serious[3]	Serious[6]	Undetected[5]	-0.081 (-0.303 to 0.141)	⊕⊕◯◯ LOW	Downgraded 2 levels: -1 for indirectness, -1 for imprecision
Visceral adipose tissue	3 (85)	Not serious[1]	Not serious[2]	Serious[3]	Serious[4]	Undetected[5]	-0.111 (-0.527 to 0.304)	⊕⊕◯◯ LOW	Downgraded 2 levels: -1 for indirectness, -1 for imprecision
Waist circumference	2 (49)	Not serious[1]	Not serious[2]	Serious[3]	Serious[4]	Undetected[5]	-0.056 (-0.601 to 0.488)	⊕⊕◯◯ LOW	Downgraded 2 levels: -1 for indirectness, -1 for imprecision
Adverse events	2 (270)	Very serious[9]	Not serious[2]	Not serious[10]	Very serious[11]	Undetected[5]	RR=1.177 (0.510 to 2.717)	⊕◯◯◯ VERY LOW	Downgraded 4 levels: -2 for very serious risk of bias (severe selective reporting), -2 for very serious imprecision (wide CI cannot exclude harm)

BIA, bioelectrical impedance analysis; BMC, bone mineral content; BMD, bone mineral density; CI, confidence interval; DXA, dual-energy x-ray absorptiometry; RR, risk ratio.

[1] 64% of studies (9/14) had a low risk of bias across all domains; the remaining 36% (5/14) had some concerns only for allocation concealment reporting.

[2] I^2=0% for all outcomes; no statistical heterogeneity detected.

[3] Serious indirectness: Interventions mechanistically distinct (whole proteins, amino acids, creatine); populations span premenopausal to postmenopausal; measurement methods varied (DXA vs BIA).

[4] Serious imprecision: 95% CI crosses null effect; sample size below optimal information size.

[5] Fewer than 10 studies per outcome; funnel plot assessment not conducted per Cochrane guidelines.

[6] Serious imprecision: 95% CI spans commonly used effect size thresholds (0.2, 0.5, 0.8), indicating substantial uncertainty regarding effect magnitude.

[7] Very serious indirectness: Total body BMD/BMC rather than clinically relevant site-specific measurements (lumbar spine, hip); intervention duration insufficient (<12 months for most studies) for meaningful bone adaptation; heterogeneous supplement types with different mechanisms.

[8] Very serious imprecision: Very small sample size (*n*=65); very wide confidence intervals; effect estimate highly uncertain.

[9] Very serious risk of bias: Only 2 of 14 included studies (14%; Chilibeck 2015, 2023) systematically reported adverse events; severe selective outcome reporting bias substantially limits confidence in safety conclusions.

[10] Not serious indirectness: Adverse events directly measured in target population; supplement heterogeneity does not compromise safety assessment.

[11] Very serious imprecision: Wide confidence interval (RR 0.510 to 2.717) cannot exclude clinically important harm (upper limit suggests potential 2.7-fold increase in adverse events); only 2 studies with small number of events.

GRADE Working Group grades of evidence:

- High quality (⊕⊕⊕⊕): Further research is very unlikely to change our confidence in the estimate of effect.
- Moderate quality (⊕⊕⊕◯): Further research is likely to have an important impact on our confidence in the estimate of effect and may change the estimate.
- Low quality (⊕⊕◯◯): Further research is very likely to have an important impact on our confidence in the estimate of effect and is likely to change the estimate.
- Very low quality (⊕◯◯◯): We are very uncertain about the estimate.

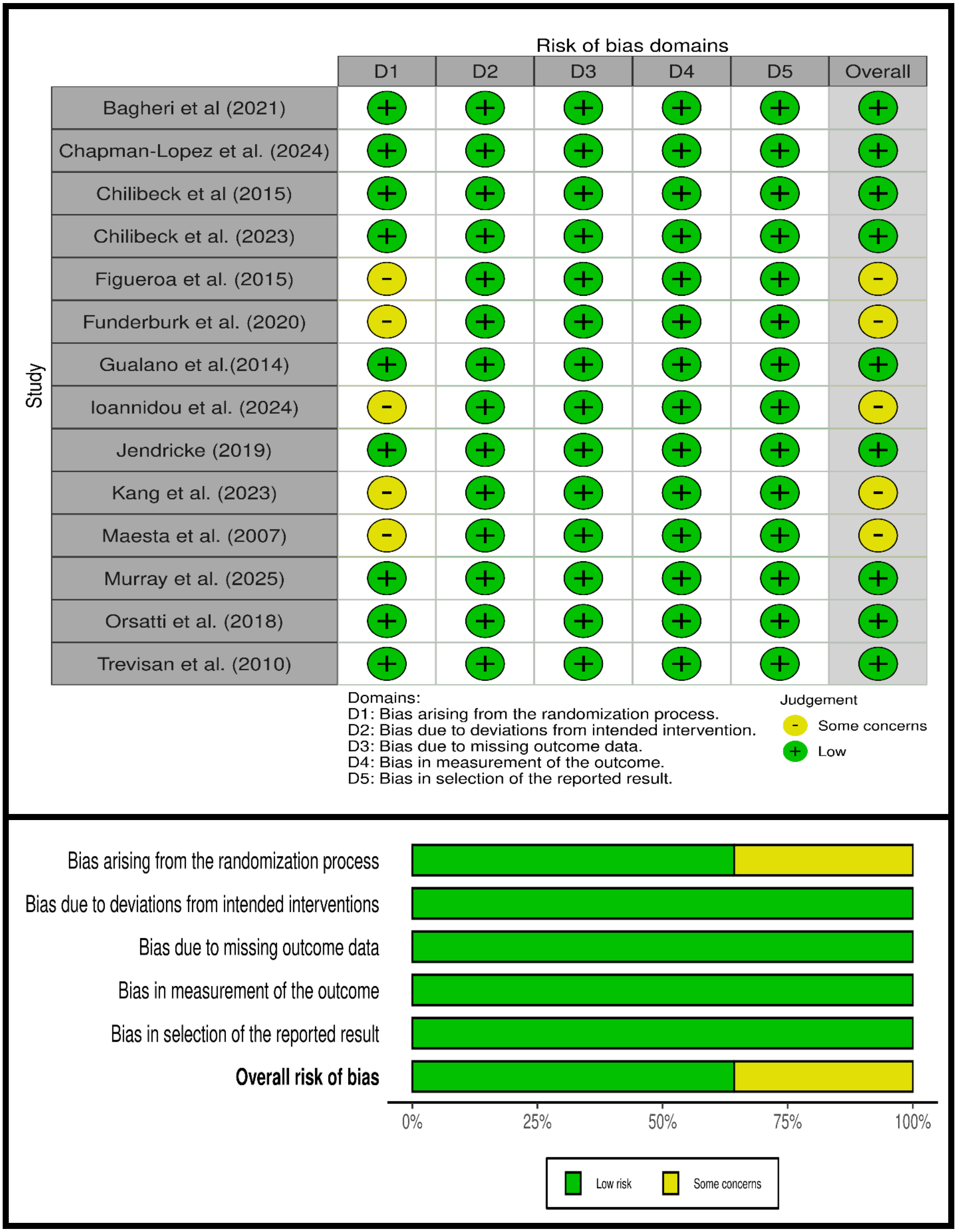

Supplementary Fig. S1 Risk of bias.

Model	Study name	Hedges's g	Standard error	Variance	Lower limit	Upper limit	Z-Value	p-Value
	Chapman-Lopez et al. (2023)	0.317	0.333	0.111	-0.335	0.969	0.953	0.340
	Gualano et al. (2014)	0.056	0.355	0.126	-0.640	0.753	0.158	0.874
Random		0.195	0.243	0.059	-0.281	0.671	0.804	0.421

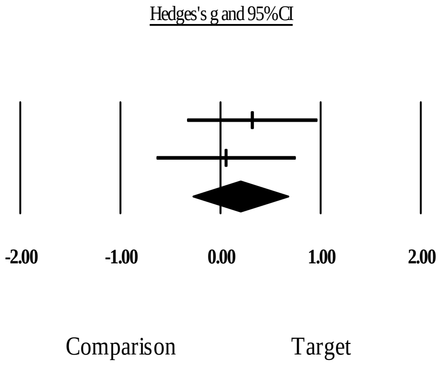

(A) Bone mineral content (kg)

Model	Study name	Hedges's g	Standard error	Variance	Lower limit	Upper limit	Z-Value	p-Value
	Chilibeck et al. (2015)	0.488	0.346	0.120	-0.191	1.167	1.409	0.159
	Chilibeck et al. (2023)	0.028	0.130	0.017	-0.226	0.282	0.215	0.829
	Gualano et al. (2014)	0.184	0.356	0.127	-0.514	0.882	0.516	0.606
	Murray et al. (2025)	0.000	0.384	0.148	-0.753	0.753	0.000	1.000
Random		0.087	0.110	0.012	-0.129	0.303	0.790	0.430

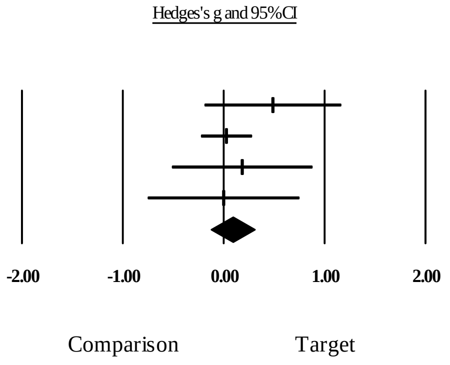

(B) Bone mineral density (g/cm^2)

Supplementary Fig. S2 Effects on bone health outcomes.

Model	Study name	Hedges's g	Standard error	Variance	Lower limit	Upper limit	Z-Value	p-Value
	Chapman-Lopez et al. (2023)	-0.008	0.330	0.109	-0.656	0.640	-0.024	0.981
	Funderburk et al. (2020)	-0.016	0.326	0.106	-0.655	0.623	-0.049	0.961
	Ioannidou et al. (2024)	-0.046	0.376	0.141	-0.782	0.691	-0.121	0.904
	Jendricke et al. (2019)	-0.010	0.226	0.051	-0.452	0.433	-0.042	0.966
	Kang et al. (2023)	-0.017	0.396	0.156	-0.792	0.758	-0.043	0.966
	Murray et al. (2025)	-0.017	0.376	0.141	-0.753	0.719	-0.045	0.964
Random		-0.016	0.130	0.017	-0.272	0.239	-0.125	0.901

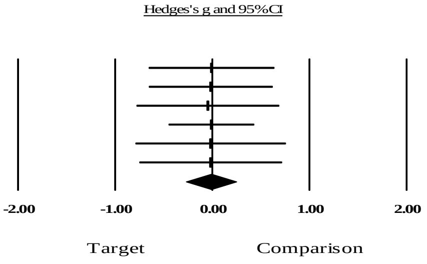

(A) Body weight (kg)

Model	Study name	Hedges's g	Standard error	Variance	Lower limit	Upper limit	Z-Value	p-Value
	Bagheri et al (2021)	-0.262	0.430	0.185	-1.106	0.581	-0.609	0.542
	Chapman-Lopez et al. (2023)	-0.002	0.330	0.109	-0.650	0.646	-0.006	0.995
	Funderburk et al. (2020)	0.000	0.326	0.106	-0.639	0.639	0.000	1.000
	Jendricke (2019)	0.000	0.226	0.051	-0.443	0.443	0.000	1.000
	Kang et al. (2023)	-0.039	0.396	0.156	-0.814	0.736	-0.098	0.922
	Maesta et al. (2007)	-0.067	0.390	0.152	-0.831	0.696	-0.173	0.863
	Murray et al. (2025)	-0.000	0.376	0.141	-0.736	0.736	-0.000	1.000
Random		-0.033	0.125	0.016	-0.279	0.212	-0.266	0.790

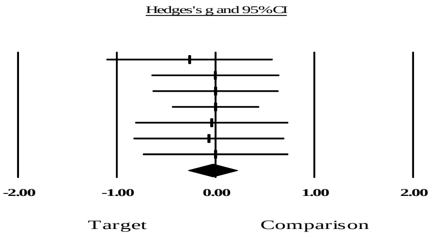

(B) Body-mass index (kg/m^2)

Model	Study name	Hedges's g	Standard error	Variance	Lower limit	Upper limit	Z-Value	p-Value
	Bagheri et al (2021)	-0.190	0.429	0.184	-1.031	0.652	-0.442	0.659
	Chapman-Lopez et al. (2023)	-0.017	0.330	0.109	-0.665	0.630	-0.053	0.958
	Figueroa et al. (2015)	0.000	0.373	0.139	-0.732	0.732	0.000	1.000
	Funderburk et al. (2020)	-0.035	0.326	0.106	-0.674	0.603	-0.109	0.914
	Ioannidou et al. (2024)	-0.102	0.376	0.141	-0.839	0.634	-0.273	0.785
	Jendricke (2019)	-0.147	0.226	0.051	-0.590	0.297	-0.648	0.517
	Kang et al. (2023)	-0.178	0.396	0.157	-0.955	0.599	-0.449	0.653
	Maesta et al. (2007)	-0.013	0.390	0.152	-0.777	0.751	-0.033	0.973
	Murray et al. (2025)	0.000	0.376	0.141	-0.736	0.736	0.000	1.000
Random		-0.081	0.113	0.013	-0.303	0.141	-0.718	0.473

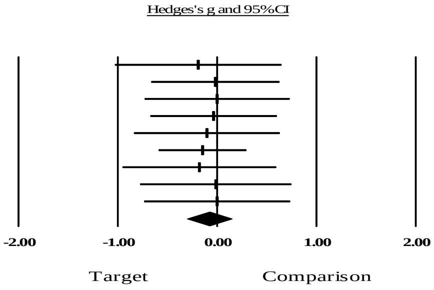

(C) Body fat (%)

Model	Study name	Hedges's g	Standard error	Variance	Lower limit	Upper limit	Z-Value	p-Value
	Chapman-Lopez et al. (2023)	-0.196	0.331	0.110	-0.845	0.454	-0.591	0.555
	Kang et al. (2023)	-0.069	0.396	0.157	-0.845	0.706	-0.174	0.862
	Murray et al. (2025)	-0.038	0.384	0.148	-0.791	0.716	-0.099	0.921
Random		-0.111	0.212	0.045	-0.527	0.304	-0.526	0.599

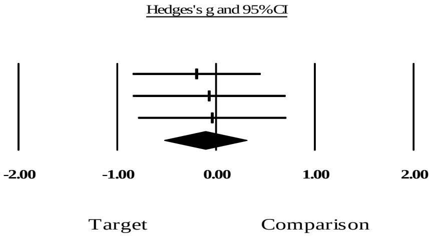

(D) Visceral adipose tissues

Model	Study name	Hedges's g	Standard error	Variance	Lower limit	Upper limit	Z-Value	p-Value
	Kang et al. (2023)	-0.023	0.396	0.156	-0.799	0.752	-0.059	0.953
	Maesta et al. (2007)	-0.089	0.390	0.152	-0.853	0.675	-0.228	0.820
Random		-0.056	0.278	0.077	-0.601	0.488	-0.203	0.839

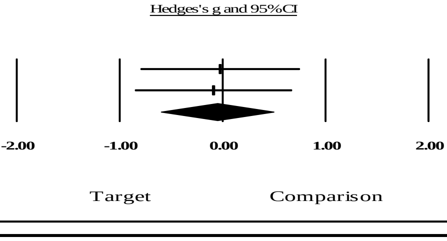

(E) Waist circumference (cm)

Supplementary Fig. S3 Effects on body composition outcomes.

Model	Study name	Risk ratio	Lower limit	Upper limit	Z-Value	p-Value
	Chilibeck et al. (2023)	0.859	0.599	1.233	-0.825	0.409
	Chilibeck et al (2015)	2.100	0.758	5.822	1.426	0.154
Random		1.177	0.510	2.717	0.381	0.703

Risk ratio and 95% CI

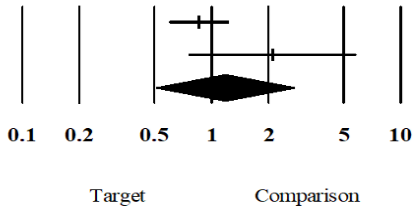

Supplementary Fig. S4 Adverse events.
